# Neutral and negative mood induction in executive tasks of working memory

**DOI:** 10.1186/s41155-021-00196-7

**Published:** 2021-10-12

**Authors:** Lívia Valenti, Ricardo Basso Garcia, Cesar Galera

**Affiliations:** 1 Department of Psychology, University of São Paulo—Ribeirão Preto School of Philosophy, Science and Literature, Av Bandeirantes 3900, Monte Alegre, Ribeirão Preto, SP CEP 14040-901 Brazil; 2 Graduate Program in Cognitive Neuroscience and Behavior, Department of Psychology, Federal University of Paraiba, João Pessoa, Brazil

**Keywords:** Mood induction, Working memory, Executive function, Verbal, Visuospatial

## Abstract

The mood induction paradigm has been an important tool for investigating the effects of negative emotional states on working memory (WM) executive functions. Though some evidence showed that negative mood has a differential effect on verbal and visuospatial WM, other findings did not report a similar effect. To explore this issue, we examined the negative mood’s impact on verbal and visuospatial WM executive tasks based on grammatical reasoning and visuospatial rotation. Participants with no anxiety or depression disorders performed the tasks before and after negative (*n* = 14) or neutral (*n* = 13) mood induction. Participants’ mood at the beginning and the end of the session was assessed by the Present Mood States List (LEAP) and word valence rating. The analyses showed changes in the emotional state of the negative group (*ps* < .03) but not of the neutral group (*ps* > .83) in the LEAP instrument. No significant differences between groups were observed in the WM tasks (*ps* > .33). Performance in the visuospatial WM task improved after mood induction for both groups (*p* < .05), possibly due to a practice effect. In sum, our findings challenge the view that negative mood modulates WM executive functions; thus, they were discussed considering the similarities and differences between studies that found negative mood effects on WM and those that did not find. Different WM tasks tap distinct processes and components, which may underlie behavioral effects of negative mood on WM tasks.

**Supplementary Information:**

The online version contains supplementary material available at 10.1186/s41155-021-00196-7.

Emotion research in the past decades has produced many exciting findings regarding the influences of emotion on our actions and beliefs, such as how we think and solve cognitive tasks and dilemmas (Kensinger & Kark, 2018; Tyng et al., 2017). The extent to which an emotional state (e.g., sad mood) influences higher cognitive functions, such as the working memory system, remains an interesting issue, particularly regarding mood impacts on executive tasks. Working memory (WM) is a limited-capacity system that temporally maintains and manipulates information in the service of cognitive tasks (e.g., problem solving; see Baddeley & Hitch, 1974; Cowan, 1988; Kane et al., 2005). In the multicomponent model (Baddeley, 2000; Baddeley & Hitch, 1974), the WM system comprises storage components (phonological loop and visuospatial sketchpad) and a central executive component that coordinates these storage components and is responsible for the control of action and attention (e.g., shifting, updating, and inhibition functions) (Miyake et al., 2000).

Previous evidence from neuroimaging studies supports the interaction between emotion and WM functions as emotional stimuli processing activates neuronal areas or circuits related to WM. For example, a critical overlap has been observed mainly in the dorsolateral prefrontal cortex, which is involved in both emotional and cognitive processing required by WM and executive tasks (Ozawa et al., 2014; Perlstein et al., 2002). Furthermore, meta-analyses studies have consistently reported that depressed people are impaired on a wide range of executive and control functions, with weak effects on memory performance (Castaneda et al., 2008; McDermott & Ebmeier, 2009; Rock et al., 2014). WM executive functions are impaired not only in clinical depression but also in subclinical conditions. According to King (2020), people who self-reported mood and emotion regulation difficulties have a reduced capacity for response inhibition in executive tasks (e.g., the Stroop, stop-signal, and go/no-go tasks) after being required to recall a sad event.

Moreover, evidence has shown a selective effect of emotion on executive and control functions, suggesting that a withdrawal state (e.g., negative mood) can lead to opposite performance patterns in verbal and visuospatial components (Gray, 2001, 2004). Gray (2001) observed an enhanced performance on a spatial *n*-back task and an impaired performance on a verbal *n*-back task in participants induced to a withdrawal state, indicating a double dissociation between spatial and verbal performance under an induced negative state. Storbeck (2012) observed a higher depletion of self-control resources (i.e., the ability to inhibit a response or a cognitive bias) when a negative mood state was combined with a verbal WM *n*-back task in comparison when a negative mood state was combined with a spatial WM *n*-back task. This finding suggests that the alignment between emotion (e.g., negative mood) and WM task demand (e.g., verbal vs. spatial) can lead to a higher or lower cognitive load, which supports the double dissociation (e.g., negative/verbal and negative/spatial) found by Gray (2001). However, it is important to note that Storbeck (2012) did not find performance differences between verbal and spatial WM tasks.

Although these findings support a relationship between emotional state and WM executive demands, some previous studies failed to find a negative mood effect on executive functions (Oaksford et al., 1996, see Experiment 3; Phillips et al., 2002; Storbeck & Maswood, 2015). For instance, Phillips et al. (2002) found no detrimental effect of negative mood on the Tower of London planning task in young people compared to older adults. Storbeck and Maswood (2015) also did not find the negative mood impact on verbal and spatial WM tasks with high executive demands.

In sum, contrasting findings were reported by studies investigating negative mood effects on WM executive control. This study aimed to further investigate the impact of negative mood on WM executive functions in verbal and visuospatial tasks. We based our research on WM tasks that place high demands on executive control (Conway et al., 2005; Kane et al., 2005; Morrison et al., 2015): a verbal task (VE) based on a grammatical reasoning task (Baddeley & Hitch, 1974) and a visuospatial task (VI) based on a rotation span task (Shah & Miyake, 1996).

The VE is based on the grammatical reasoning task devised to investigate the executive component of WM (Baddeley, 1968; Baddeley & Hitch, 1974) and requiring cognitive flexibility and reasoning (Morrison et al., 2015) with reliability of .94 (Tabatabaee-Yazdi, 2018). It requires the ability to rapidly and accurately judge confusing grammatical sentences about memorized stimuli. Furthermore, the VE task has been explored in a previous Brazilian study to investigate fluid reasoning (Bedi et al., 2021). The VI task is based on the rotation span task devised by Shah and Miyake (1996). It is a complex span task that requires executive control to coordinate between concurrent tasks, as it requires temporarily memorizing visuospatial information while evaluating stimuli characteristics (Case et al., 1982; Conway & Engle, 1996; Conway et al., 2005). Furthermore, Miyake et al. (2001) findings have demonstrated through confirmatory factor analysis that the VI task implicates executive functioning, correlated to other executive tasks (e.g., Tower of Hanoi).

Based on Gray (2001) findings, we expected that a negative mood would impair performance on the VE task and improve performance on the VI task. We consider that investigating this issue may contribute to better understanding the cognitive effects of negative context and mood in healthy individuals.

## Method

### Participants

Twenty-seven volunteers from a Brazilian university (15 females, *M* = 24 years, *SD* = 3.37) participated in the study. We randomly assigned the students to either a neutral (*n* = 13) or a negative mood induction group (*n* = 14). The sample size was estimated by a power analysis performed in G*Power (Faul et al., 2007) for a repeated-measures ANOVA with within-between interaction, as this study had a between-subjects factor (neutral vs. negative mood induction) with four measures in the within-subjects factors task (VE and VI) and induction moment (pre- vs. post-induction). Considering a within-between interaction of effect size *f*(*U*) = .50 (*η*^2^ = .20 as in SPSS), power (1 − *β*) = .90, and *α* = .05, a total sample size of 24 participants was estimated.

We ensured that participants entering the study met specific criteria regarding depression and anxiety symptoms. The participants were evaluated with the Beck Anxiety Inventory (Beck et al., 1988) and Beck Depression Inventory-II (BDI-II, Beck et al., 1996). All of the participants had scores below cut-off values in the BDI (17 points) and the BAI (10 points) (Cunha, 2001). None of the participants was referred to as having a mood disorder. The participants signed informed consent forms, and the local Research Ethics Committee approved the study. During the recruitment of participants, a total of 20 individuals did not meet the inclusion criteria.

### Materials and stimuli

Stimuli were presented using the E-prime software (Schneider et al., 2002). For the visuospatial and verbal tasks, stimuli were letters presented in Arial Black font, which occupied approximately one degree of visual angle on a uniform white background. For the mood induction, stimuli were images from the International Affective Picture System (IAPS) (Lang et al., 1999) with Brazilian norms (Ribeiro et al., 2004) and pieces of music (details described in the following topic). The supplemental archive shows the number codes of the IAPS images selected for each induction group (negative vs. neutral images).

#### Mood induction

The negative and neutral mood induction comprised 51 images with negative valence (valence: *M* = 1.73, *SD* = 1.25; arousal: *M* = 7.55, *SD* = 1.67) and 51 images with neutral valence (valence: *M* = 5.10, *SD* = 1.64; arousal: *M* = 4.45, *SD* = 1.89). The images were presented in two blocks of 27 images for 2.4 min (5.3 s per image) (Baddeley et al., 2012), simultaneously with 2.4 min of the music Symphony 5 - Adagietto by Mahler (negative induction) (Storbeck & Clore, 2005) or “Pure Arctic Wind” music from the “Nature Sounds-Arctic Wind” compilation (neutral induction).

#### Mood evaluation

The participants completed two instruments at the beginning and the end of the experimental session: (1) a list of mood expressions adapted from the Present Mood States List or LEAP (“Lista de Estados de Ânimo Presentes – LEAP,” Engelmann, 1986) and (2) a list of words with neutral, positive, and negative valence (Oliveira et al., 2013). The LEAP comprised 40 expressions classified in 12 factors of mood states. In Engelmann (1986, 2002) studies, the LEAP instrument was based on factorial analysis of the mood expressions, according to the arousal and valence attributed to them by a sample of Brazilian participants. Other studies have used the LEAP instrument for evaluating emotional states in nurses (Bueno et al., 2003) and volleyball athletes (Bueno & Di Bonifácio, 2007). However, none of these studies reported reliability and validity for the LEAP.

Each expression was displayed in the center of the monitor for 1500 ms, followed by a 5-point Likert scale, representing the intensity of the participant’s emotional state: 1—very weak, 2—weak, 3—average, 4—present, 5—very present. Participants judged each expression according to their mood state intensity at the moment the sentence was being read. The supplemental archive shows the LEAP list of 40 expressions.

The list of words was based on previous studies that measured mood by evaluating words’ valence (Baddeley et al., 2012;Fachinello, 2018 ; Fachinello et al., 2021). Baddeley et al. (2012) examined the negative mood effect in evaluating affective information by measuring judgment ratings of valenced stimuli-like words. They found that negative mood resulted in substantial changes in valence ratings by the participants, who perceived negative stimuli as being more negative. This may be considered an implicit measure assessing the participant’s mood and the effect of induction. In a previous Brazilian study (Fachinello, 2018; Fachinello et al., 2021), a shorter form of the word valence rating task (Baddeley et al., 2012) was used for investigating the mood induction effect on WM, and the results showed that ratings of negative words significantly changed after mood induction.

The words were selected from a 908 Portuguese word database classified according to arousal and valence (Oliveira et al., 2013). Two different lists were used (Fachinello, 2018; Fachinello et al., 2021), each with 15 words and five words for each valence (i.e., neutral, positive, and negative). They were presented at the beginning and the end of the experimental session. In total, 10 neutral words (valence: *M* = 5.26, *SD* = 1.87; arousal: *M* = 4.38, *SD* = 1.85), 10 positive words (valence: *M =* 8.17, *SD* = 1.63; arousal: *M =* 3.10, *SD =* 2.46), and 10 negative words (valence: *M =* 1.85, *SD* = 1.86; arousal: *M =* 7.40, *SD =* 1.87) were used. Each word was displayed in the center of the monitor screen for 1000 ms and was followed by an 8-point Likert scale with the 1-point value referred to as “Extremely Negative” and 8-point value as “Extremely Positive.” The supplemental archive shows both the word lists.

#### VE task

This task is based on the grammatical reasoning task from the Baddeley and Hitch (1974) study. In this task, the participants were required to memorize a pair of letters (i.e., “AB”) and were required to answer whether a given statement about the letters was “true” or “false.” For example, if the pair “AB” was followed by the statement “A follows B,” the correct answer would be “false.” The task comprised 32 statements about combinations between the letters “A” and “B” (either “AB” or “BA”), based on the following elements: (1) “precedes” or “follows,” (2) active or passive voice, and (3) positive or negative sentence. For example, given the pair “AB,” the statements “A is followed by B” and “B is not followed by A” are both true. There were 16 practice trials at the beginning of the experimental session.

#### VI task

The Shah and Miyake (1996) task was designed to simultaneously assess the ability to process and store visuospatial information. In each trial of this task, the participant saw a sequence of two capital letters or their mirror images, presented one by one, each rotated differently. The participant’s task was to answer whether each letter was presented as “normal” or “mirrored” while memorizing the orientation (using the top of the letter as reference) of each letter in the sequence. At the end of the sequence, the participant’s task was to remember the letter orientations in the correct order they were presented. The letters were selected from a set of five letters (F, P, R, L, and J) and were presented in the center of the screen. After the letter sequence presentation (i.e., with normal or mirror direction), a diamond-shape grid with squares (i.e., buttons for mouse clicks) comprising eight squares of 1.14° × 1.14° degrees of visual angle was displayed. The eight squares were placed in 45° increments representing the possible locations of the top of the letters. Each letter was presented in a normal or mirror image in one of seven possible orientations, in 45° increments except by the standard upright position, with a total of 70 possible combinations (5 letters × 7 orientations × 2 directions).

### Procedure

**Fig. 1:**
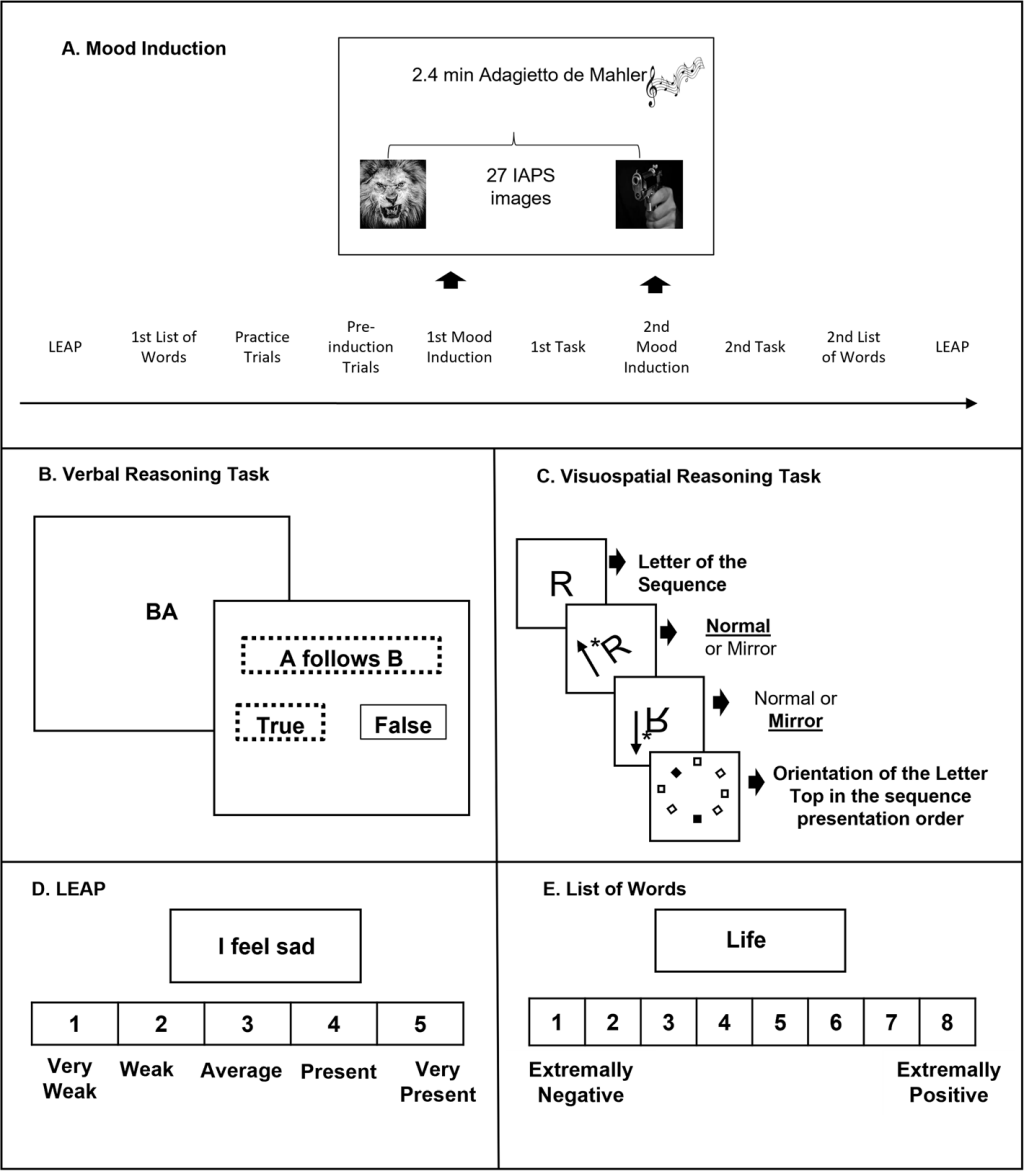
Sequence of events in a trial. Note: (**A**) Illustration of the sequences of events in the negative group, (**B**) verbal task, (**C**) visuospatial task (e.g., sequence of two letters), (**D**) LEAP, and (**E**) list of words

Previous to the experimental session, each participant was evaluated by the BDI-II and BAI. The participants’ mood was evaluated by using the LEAP and the list of words for valence rating at the beginning and end of the experimental session. In contrast with Baddeley et al. (2012) study, our experiment had cognitive tasks after mood induction and not the word valence rating task, as our focus was investigating the mood effect on the memory tasks. For this reason, the post-induction word list was presented after the memory tasks and not immediately after the mood induction procedure. After the initial mood evaluation, the participants performed, in counterbalanced order, the baseline trials of the VE and VI tasks. The mood induction occurred after the pre-induction trials and was followed by two blocks of post-induction trials (VE and VI tasks). At the end of the experimental session, participants completed the two mood evaluation instruments again. Figure 1A describes the trial sequence, Fig. 1 B and C depict the tasks, and Fig. 1 D and E depict the mood evaluation instruments.

#### VE task

In each trial, a pair of letters was presented for 500 ms and was followed by a mask (i.e., the pair of letters “XX”) for 250 ms. After the mask, a sentence describing the pair order was presented on the screen (e.g., “A precedes B”) for 8000 ms. Below the sentence, two boxes with the words “True” and “False” were presented on the left and right sides, respectively. The participant was instructed to press the mouse’s left button if the sentence was “True” or press the mouse’s right button if the sentence was “False.” If the participant’s response was delayed and exceeded the time available, a null response was punctuated and the feedback “No Response” in red would appear on the screen regardless the type of trial (i.e., practice, pre-induction, and post-induction trials). The participant performed 16 practice trials, 10 pre-induction trials, and two practice trials before the 32 post-induction trials. In the practice trials, responses were followed by feedback (blue cross or red cross for correct and incorrect responses, respectively).

#### VI task

In each trial, the participant was presented with a sequence of two letters and was required to answer whether each letter image was normal or mirrored. Each letter remained on the screen for a maximum of 2200 ms. The following letter appeared on the screen after an interval of 250 ms. After the entire sequence of letters was presented, a diamond-shaped grid was displayed on the screen with eight square “buttons” marking the seven possible letter orientations (plus the top vertical). The participant’s task was to use the mouse to click on the corresponding buttons for indicating each letter orientation in its order of appearance. After the participant had clicked on the appropriate number of buttons, the grid was removed from the screen, and the participant had to press the space key to start the next trial.

At the beginning of the VI task, participants performed 16 training trials in which they had to identify only the answer whether the letter was in normal or mirrored direction, and 16 trials in which they had to answer whether the letter had normal or mirrored direction, and to indicate their orientations on the grid. After this training, the participant performed 10 pre-induction trials (i.e., baseline performance). After the mood induction, the participants performed 32 trials, the first two considered training trials.

### Data analysis

The proportion of correct answers was computed for each trial. In the visuospatial reasoning task, a correct trial was computed when the participant correctly answered both the direction (i.e., mirrored or normal) and orientation (i.e., the top of each letter). The data were submitted to mixed repeated-measures ANOVAs with induction group (negative and neutral) as a between-subjects factor, task (i.e., VE and VI tasks), and moment (i.e., pre- and post-induction trials) as within-subjects factors.

In the analyses of mood ratings, we performed ANOVAs with induction group as between-subjects and pre-induction and post-induction moment as within-subject factors for the average ratings of words according to their valence and, separately, for the list of expressions (i.e., LEAP). For all the analyses, the significance level was set at .05, partial Eta squared (*η*^2^_p_) effect sizes were computed, and post hoc analyses and *t* tests using Bonferroni’s correction were carried out as necessary. To better explore the differences due to the negative mood induction, we analyzed only expressions from the LEAP list related to extremely negative and positive feelings. Four expressions related to negative feelings (“I feel sad,” “I am scared,” “I am angry,” “I am disgusted”) and one positive expression (i.e., “I am happy”) were examined. Other expressions were not included in the analysis because they involved a more complex state of emotion or feeling (e.g., “I feel surprised,” “I feel sexual attraction”). Thus, our criteria provided an examination of a negative mood instead of broader emotional states. The supplemental material shows the database (i.e., excel files) used in the study.

## Results

### WM tasks

The 2 induction group × 2 task (VE and VI tasks) × 2 moment (pre- vs. post-induction) repeated measures ANOVA revealed a main effect of moment, *F*(1, 25) = 4.85, *p* = .037, *η*^2^_p_ = .16, given that participants performed better in the post-induction trials (*M* = .68, *SE* = .03) than in the pre-induction trials (*M* = .63, *SE* = .04). The main effect of group *F*(1, 25) = .02, *p* =.89, *η*^2^_p_ =.001, and task, *F*(1, 25) = .69, *p* =.41, *η*^2^_p_ = .027, were not significant. The mean scores of pre- and post-induction trials for the VE and VI tasks and each group are shown in Table 1.

**Table 1:** The mean scores of pre-post induction trials for VE and VI tasks in each group.

	Verbal task		Visuospatial task	
	Pre-induction	Post-induction	Pre-induction	Post-induction
	Mean (*SE*)	Mean (*SE*)	Mean (*SE*)	Mean (*SE*)
Negative Group	.69 (.05)	.71 (.04)	.57 (.09)	.66 (.08)
Neutral Group	.68 (.05)	.64 (.04)	.57 (.10)	.71 (.08)

The interaction between moment and task was significant, *F*(1, 25) = 5.38, *p* = .03, *η*^2^_p_ = .17, but the other interactions were not significant (*Fs* < 1, *ps* > .33). To explore the significant interaction, we examined the participants’ performance in pre- and post-induction trials separately for each task. The results revealed a significant difference between pre-induction and post-induction trials for the VI task, *F*(1, 25) = 8.70, *p* = .007, *η*^2^_p_ =.26, with higher performance in the post-induction trials than for pre-induction trials. In contrast, there was no significant difference between moments for the VE task, *F*(1, 25) = .07, *p* = .79, *η*^2^_p_ =.003.

### Mood ratings

Tables 2 and 3 show participants’ mean ratings for the evaluation of expressions and words for each induction group.

**Table 2:** The mean scores of participants for the evaluation of expressions in each group

Expressions	Negative group		Neutral group	
	Pre-induction	Post-induction	Pre-induction	Post-induction
	Mean (*SE*)	Mean (*SE*)	Mean (*SE*)	Mean (*SE*)
I am happy	3.71 (.16)	2.79 (.21)	3.00 (.25)	2.85 (.30)
I am scared	2.00 (.23)	2.57 (.34)	2.31 (.33)	2.23 (.26)
I am disgusted	1.07 (.07)	2.71 (.27)	1.31 (.24)	1.38 (.27)
I feel sad	1.93 (.29)	3.00 (.26)	2.46 (.29)	2.62 (.31)
I am angry	1.79 (.28)	2.07 (.27)	2.92 (.38)	2.77 (.43)

**Table 3:** The mean scores of participants for the evaluation of words in each group

Words	Negative group		Neutral group	
	Pre-induction	Post-induction	Pre-induction	Post-induction
	Mean (*SE*)	Mean (*SE*)	Mean (*SE*)	Mean (*SE*)
Negative words	2.03 (.18)	1.93 (.20)	1.75 (.19)	1.60 (.20)
Neutral words	5.09 (.22)	4.59 (.15)	4.75 (.22)	4.51 (.16)
Positive words	7.69 (.11)	7.47 (.18)	7.12 (.11)	7.05 (.19)

#### List of expressions (LEAP)

The 2 induction group (negative vs. neutral) × 2 moment (pre- vs. post-induction) × 5 expression (happy vs. scared vs. disgusted vs. sad vs. angry) repeated measures ANOVA revealed a main effect of moment, *F*(1, 25) = 8.22, *p* = .008, *η*^2^_p_ = .25, given that ratings were higher in the post-induction (*M* = 2.50, *SE* = .14) than in the pre-induction (*M* = 2.25, *SE* = .10). The main effect of expression was also significant, *F*(4, 100) = 11.16, *p* < .001, *η*^2^_p_ = .31. Pairwise comparisons showed that participants judged the emotional mood “I am happy” with higher intensity than the other expressions (all *ps* < .04), except for the expression “I am sad” (*p* = .57) and “I am angry” (*p* = .19). Participants rated the emotional state “I am disgusted” with lower intensity than the other expressions (all *ps* < .01). The other differences were not significant (all *ps* =1). The main effect of induction group was not significant, *F*(1, 25) = .009, *p* = .93, *η*^2^_p_ < .001.

The two-away interactions between moment and induction group, *F*(1, 25) = 10.39, *p* = .004, *η*^2^_p_ = .29; expression and induction group, *F*(4, 100) = 3.17, *p* = .017, *η*^2^_p_ = .11; moment and expression, *F*(4, 100) = 11.06, *p* <.001, *η*^2^_p_ = .31 were significant. The three-way interaction between moment, expression, and induction group, *F*(4, 100) = 6.99, *p* <.001, *η*^2^_p_ = .22, was also significant. In this section, we only explored the effects of interaction between induction groups (negative vs. neutral) across pre- and post-induction for each expression. The other interactions were not considered due to the study’s focus on analyzing the effects of mood induction for both groups.

Further analyses showed that participants in the negative group rated with higher intensity the mood state “I am disgusted” (*p* < .001) and “I feel sad” (*p* = .03) after the induction. In contrast, the emotional state “I am happy” was rated with lower intensity (*p* = .005) in the post-induction moment. There were no significant differences between pre- and post-induction expression ratings for the neutral group (all *ps* > .83). The rating difference between groups for each expression in the pre-induction trials (all *ps* > .49) and post-induction trials was not significant (all *ps* > .18).

#### List of words

The 2 induction group (negative *vs*. neutral) × 2 moment (pre- *vs*. post-induction) × 3 valence word (negative *vs*. neutral *vs*. positive) repeated measures ANOVA revealed a main effect of induction group, *F*(1, 25) = 10.54, *p* = .003, η^2^_p_ = .30, given the overall higher word ratings for the negative (*M* = 4.80, *SE* = .07) than the neutral group (*M* = 4.46, *SE* = .07). There was a significant main effect of the moment, *F*(1, 25) = 7.86, *p* = .010, η^2^_p_ = .24, with higher scores in the pre-induction (*M* = 4.74, *SE* = .06) than in the post-induction (*M* = 4.52, *SE* = .06). Also, the main effect of valence words rating was significant, *F*(2,50) = 561, *p* < .001, η^2^_p_ = .96, and pairwise comparisons revealed that participants rated the positive words with higher scores (*M* = 7.33, *SE* = .08) than negative (*M* = 1.82, *SE* = .12) and neutral words (*M* = 4.73, *SE* = .11), and the negative words were rated with lower scores than the neutral ones (all *ps* < .001). These results suggest that the words were evaluated according to their affective valence range (e.g., 1 = extremely negative, 8 = extremely positive). The other interactions were not significant (*Fs* < 1, *ps* > .35)

## Discussion

The current study investigated the negative mood effect on working memory, mainly on executive function tasks involving visuospatial and verbal domains in healthy individuals. Participants performed working memory tasks based on the grammatical reasoning task (Baddeley & Hitch, 1974), VE task, and the rotation span task of Shah and Miyake (1996), VI task, after a neutral or a negative mood induction. Our results showed that both groups had better performance in post-induction than in pre-induction trials in the VI task, but no performance improvement was observed in the VE task. The ratings of mood-related LEAP expressions significantly changed from the initial to the final evaluation for the negative mood induction group, but not for the neutral group, indicating that negative mood induction was effective. However, a similar effect was not observed for word ratings, indicating that this task was not sensitive to capture mood changes. In sum, the results showed no change in WM performance due to a negative mood induction but only a learning or practice effect in the VI task, as both groups presented a better performance in the post-induction trials.

The present study results contrast with previous findings regarding a moderating role of negative mood on WM performance. In particular, we did not observe a differential mood effect on verbal and visuospatial WM modalities as reported in previous studies (Gray, 2001; Gray et al., 2002; Li et al., 2010; Storbeck & Watson, 2014). It is interesting to note that previous evidence has demonstrated an impact of negative mood in spatial (Gray, 2001) and verbal working memory (Osaka et al., 2013). However, like our study, some studies did not find a negative mood effect on WM tasks with high executive demands in the verbal (Miller et al., 2018), visuospatial (Palmiero et al., 2016), or both domains (Storbeck & Maswood, 2015). For instance, Gray (2001) found that participants induced into a negative mood were better in a spatial *n*-back task than in a verbal *n*-back task. In contrast, Storbeck and Maswood (2015) found no negative mood effects in a WM operation span task (i.e., spatial and verbal task). Each of the findings will be discussed in turn to explore our results for each task domain (e.g., verbal and visuospatial).

### Mood effects on the VI task

In contrast with Gray’s (2001) findings, we found no support for the hypothesis that negative mood improves performance on visuospatial WM tasks. Compared with Gray’s study, an explanation for the lack of the negative mood effect in this task might be related to task differences between studies (Ribeiro et al., 2018). For instance, our study failed to find a negative mood effect on a visuospatial WM executive function task based on the Shah and Miyake (1996) complex span test. In Gray (2001), the negative mood enhanced performance on a spatial *n-*back task. Our results are consistent with those found by Storbeck and Maswood (2015) that investigated the effect of positive and negative mood on a WM task involving both information storage and executive control processing, like the VI task used in our study. These authors found no negative mood effects on visuospatial and verbal complex span tasks that involved the maintenance of locations or words while performing a concurrent math problem-solving task. Like the Storbeck and Maswood (2015) study, our VI task was based on a storage-plus-processing test in which participants engage in the memorization of stimuli (i.e., the orientation of letters or the letter sequence) while also performing a concurrent task (i.e., indicating whether a letter was normal or mirrored, or processing the meaning of a sentence about the letter sequence).

Though negative mood seems to enhance retrieval of visual information in an *n-*back task (Gray, 2001) or boost visual memory resolution in a color recall task (Xie & Zhang, 2016), other studies have reported no effects on operation span tasks (Jonkman et al., 2017; Miller et al., 2018; Storbeck & Maswood, 2015). Thus, contrasting evidence of negative mood effect on WM might be due to different WM measures tapping the WM system’s distinct processes or components. For instance, two relevant studies (Kane et al., 2007; for a review, see Redick et al., 2013) support the assumption that both complex span and *n*-back tasks seem to tap different aspects of WM functioning. First, the *n*-back or recall tasks may trigger an update and maintenance of information which do not require the same degree of executive control as the memory span tasks (Kane et al., 2007). This evidence is consistent with a meta-analysis review that indicated weak correlations between these two types of tasks (Redick et al., 2013). In addition, the lack of similarity between such tasks is often demonstrated in WM-training studies that usually investigate whether practice on one type of WM measure (i.e., *n-*back) influences the performance of another WM task (i.e., operation span task). For instance, training on an *n-*back task improves performance in simple span tasks after a training phase, but not in complex WM span tasks (Redick et al., 2013).

However, divergences between WM measures cannot entirely explain the inconsistent findings of mood effects on WM. First, this assumption is contrasted by reports of a positive correlation between complex span and *n-*back tasks that manipulated visuospatial content, although the lowest correlation was observed when both tasks used verbal information (Redick et al., 2013; Schmiedek et al., 2009). Furthermore, studies using *n*-back or recognition tasks have also failed to find WM performance changes related to a negative mood induction (Souza et al., 2021; Storbeck, 2012). For example, in a recent study, Souza et al. (2021) investigated the effect of negative mood on WM precision (i.e., a qualitative aspect) and WM capacity (i.e., a quantitative aspect) in a similar visual recognition task used by Xie and Zhang (2016). In contrast to Xie and Zhang’s (2016) findings, Souza et al. (2021) did not find a negative mood effect across six experiments. Thus, it seems that besides divergences between WM paradigms, there must be other possibilities to explain the lack of mood effect. However, this methodological issue remains an important issue to further studies.

### Mood effects on the VE task

In contrast to our initial hypothesis, our findings revealed no negative mood effect for the VE task. It also contrasts with Gray’s (2001) findings that negative mood impairs verbal WM executive tasks. The reasoning component in our VE task paradigm might possibly be a crucial point to explain these differences between studies. For example, our results are supported by previous studies that investigated mood induction on syllogism reading tasks (Rodríguez-Gómez et al., 2019; Smith et al., 2014) that found no effects of emotional state on behavioral performance. Syllogism reading tasks pose a high executive load on WM, as our VE task.

In Rodríguez-Gómez et al. (2019), participants performed a syllogism-reading task after induced negative, neutral, and positive moods. Participants read a major premise (e.g., “All men are mortal”), a minor premise (e.g., “Juan is a man”), and the conclusion (e.g., “Juan is mortal”). Similar to our task (e.g., the pair “AB” and the statement “A is followed by B,” this sentence being true for the given pair), the aim was to judge whether the conclusion was logically valid or not. Behavioral and electrophysiological responses were used to measure mood effects. Findings showed that neither positive nor negative mood significantly affected behavioral performance, although electrophysiological results revealed that a negative mood state influenced logical processing. Smith et al. (2014) also found no detrimental effect on performance for syllogistic reasoning tasks, but neuroimaging data (e.g., fMRI scanning technique) showed that positive and negative states did have dissociable effects on the underlying neural mechanisms involved in reasoning.

The fact that these studies found neural level differences related to mood in tasks that recruit executive processes (e.g., reasoning component), despite a lack of behavioral difference, suggests that mood affects the processing in some levels of the cognitive system. Thus, the absence of behavioral effects in our study (i.e., performance impairment or improvement) does not exclude the possibility of an interaction between mood and WM executive function. Many studies have shown subtle mood effects on behavioral performance but robust brain activation differences (Aoki et al., 2011; Figueira et al., 2017; Li et al., 2010).

It is important to note that additional measures are relevant to assess participants’ emotional states effects on cognition. For example, electrophysiological evidence supports strong mood effects even in the absence of behavioral evidence (Perlstein et al., 2002; Renner et al., 2017). Our study was restricted to the behavioral level and did not explore effects at the neural level. Therefore, further research on this issue may be warranted.

### Limitations

One possible criticism of our study is that our participants may not have responded in the same manner to the negative emotional state. Individual differences might have contributed to the efficient management of mood effects, thus preventing a WM impairment. According to previous studies, individual differences in the regulatory process (Figueira et al., 2017; Szasz et al., 2016; Totterdell & Parkinson, 1999) or cognitive capacity (Chuderski, 2015; Fairfield et al., 2015) may offer a partial explanation for the diverging findings. For instance, Szasz et al. (2016) investigated how regulation strategies that attenuate negative states (e.g., anger and sadness) affect WM tasks’ decision-making performance. The authors found that adaptive strategies that modulated negative moods promoted a better capacity to make decisions. Regarding individual differences in cognitive processing, previous studies have shown a correlation between intelligence quotient or fluid intelligence and mood states during working memory tasks (Chuderski, 2015; Fairfield et al., 2015), suggesting that cognitive capacity can mediate the relationship between mood and working memory. Future studies should account for these individual differences to better understand the negative mood effect on executive WM tasks.

Furthermore, another limitation is that our second mood measure, the list of words, was not sensitive to detect mood changes. We did not replicate Baddeley et al. (2012) and Fachinello et al. (2021) findings, which showed an influence of negative mood on word evaluation. There were, however, some procedural differences between our studies. In our experiment, we presented the list of words with 15 words each in two moments, before the practice trials (i.e., pre-induction) and after the participants accomplished the tasks (i.e., post-induction). Arguably, this interval between the mood procedure and a list with fewer items for evaluation could have weakened the magnitude of the mood induction effect on this task and its sensitivity to detect mood fluctuations. However, it is important to note that we used the same lists of a previous Brazilian study that found significant differences in mood as measured by the list of words with 15 words each (Fachinello, 2018; Fachinello et al., 2021).

The list of words is an implicit measure aimed at detecting mood changes by analyzing how emotional words have their valence rated. In contrast, the list of expressions (i.e., LEAP list) involves an explicit response of the participant toward her or his emotional state (e.g., rating a feeling of sadness or happiness). In this case, an implicit measure might entail more items (i.e., trials) at different moments of the experiment to better assess mood variations than an explicit mood measure. Therefore, future studies should consider the list of words with adequate adjustments to the experimental paradigm to better assess mood induction.

Likewise, one interpretation for this limitation is that participants have not been mood-induced in our study. However, if this were the case, we would expect no mood changes as measured by the LEAP in the negative group, but we found a robust negative mood state after the induction as indicated by mood-related items from the LEAP self-rated scale. Previous studies have used self-rated scales to assess mood induction effectiveness (Joseph et al., 2020) and found consistent evidence that accounts for this measure’s validity. Therefore, we see no reasons to doubt the occurrence of negative mood induction in our study, as one might have argued based on the non-significant effect on WM performance.

## Conclusions

In summary, our study found no effect of negative mood on verbal and visuospatial WM tasks with high executive demands in healthy participants. However, the absence of improvement or impairment of performance does not mean a lack of interaction between emotion and cognition. Previous findings consistently demonstrated that depressed patients have a deficiency in executive and control functions (Bellaera & von Mühlenen, 2017; King, 2020), suggesting an evident influence of emotion on the WM system, although some contrasting findings were observed in the literature and may be related to methodological discrepancies between tasks and studies (Ribeiro et al., 2018).

Although we failed to replicate a negative mood effect reported in Gray (2001), relevant factors should be considered mediators to this lack of emotion-cognition interaction. First, the WM is a multifaceted system that relies on multiple processes (e.g., updating, inhibition, encoding, maintenance, recall, recognition) and components (e.g., verbal, visuospatial, tactile). Thus, different WM tasks might tap distinct processes or components. Second, a multi-measure approach, such as the fMRI technique and event-related potentials, may be important to assess mood effects on WM. Third, elaborative strategies and individual differences in cognitive capacity account for mood regulation that would prevent an impairment of the negative emotion on WM. Taken together with this evidence, it is crucial as a next step to investigate these assumptions to shed light on emotional states’ influence on the decision-making of healthy individuals.

## Supplementary Information

**Additional file 1.** Induction group (Negative vs. Neutral) x Tasks Accuracy (Visuospatial vs. Verbal) x Moment (Pre-induction vs. Post-induction). EscalaLikert.: Induction group (Negative vs. Neutral) x Moment (LEAP1 vs. LEAP2) x Expressions (Happy vs. Scared vs. Disgusted vs. Sad vs. Angry). EscalaLikert.: Induction group (Negative vs Neutral) x Moment (List1 vs List2) x Valence Words (Negative vs Neutral vs Positive)

**Additional file 2.** Lists of Words

**Additional file 3.** LEAP Expressions

**Additional file 4.** IAPS Images

## Data Availability

All data generated or analyzed during this study are included in this published article (Database—Sup. Material_2021). In addition, the mood evaluation instruments used in this study (e.g., list of expressions; list of words) are also included (LEAP Expressions—Sup. Material; Lists of Words—Sup. Material).

## Abbreviations

BAI: Beck Anxiety Inventory
BDI: Beck Depression Inventory-II
LEAP: Present Mood States List
VE: Verbal task
VI: Visuospatial task
WM: Working memory
