# Supplementary material for: Neutral and negative mood induction in executive tasks of working memory
Source: Psicol Reflex Crit. 2021 Oct 12;34:31. doi: 10.1186/s41155-021-00196-7 (PMC8511203; doi:10.1186/s41155-021-00196-7)
Supplement: Supplementary file 2 — Additional file 2. Lists of Words [file 41155_2021_196_MOESM2_ESM.pdf]

Lists of Words

LIST 1			LIST 2		
Positive	Neutral	Negative	Positive	Neutral	Negative
Alegria	Básico	Doença	Abraço	Asfalto	Dor
Cura	Cesto	Ladrão	Conquista	Caneta	Fracasso
Harmonia	Farelo	Tristeza	Vida	Estado	Pesadelo
Bondade	Lápis	Agressão	Aconchego	Habitante	Corrupção
Paraíso	Bandeira	Sofrimento	Férias	Bolso	Mal-estar
