# Supplementary material for: Neutral and negative mood induction in executive tasks of working memory
Source: Psicol Reflex Crit. 2021 Oct 12;34:31. doi: 10.1186/s41155-021-00196-7 (PMC8511203; doi:10.1186/s41155-021-00196-7)
Supplement: Supplementary file 3 — Additional file 3. LEAP Expressions [file 41155_2021_196_MOESM3_ESM.pdf]

LEAP

1. Sinto-me humilhado(a)
2. Sinto-me culpado(a)
3. Sinto-me triste
4. Estou com medo
5. Estou sem graça
6. Sinto raiva
7. Estou com vergonha
8. Estou com inveja de alguém
9. Estou com nojo
10. Sinto ciúme de alguém
11. Sinto uma necessidade
12. Estou tomando cuidado
13. Tenho pena de alguém
14. Estou alegre
15. Estou cansado(a)
16. Estou com sono
17. Estou cheio
18. Sinto uma obrigação
19. Estou com fome
20. Sinto um desejo
21. Estou com esperança
22. Estou gostando de alguém
23. Sinto-me interessado(a)
24. Tenho pena de alguém
25. Sinto uma admiração por alguém
26. Sinto-me orgulhoso(a)
27. Sinto-me surpreso(a)
28. Acabo de levar um susto
29. Acho algo estranho
30. Estou refletindo
31. Estou com frio
32. Faço pouco caso de alguém
33. Acho algo gozado
34. Estou aceitando alguma coisa
35. Estou com calor
36. Sinto atração sexual por alguém
37. Sinto-me calmo(a)
38. Estou conformado(a)
39. Estou com sede
40. Sinto saudade de alguém
