# Supplementary material for: Neutral and negative mood induction in executive tasks of working memory
Source: Psicol Reflex Crit. 2021 Oct 12;34:31. doi: 10.1186/s41155-021-00196-7 (PMC8511203; doi:10.1186/s41155-021-00196-7)
Supplement: Supplementary file 4 — Additional file 4. IAPS Images [file 41155_2021_196_MOESM4_ESM.pdf]

IAPS Images

Negative Images	*Neutral Images*[a]
3530	7710
9250	7705
9910	7207
9920	7190
9050	7175
3000	7140
6300	7130
9433	7095
9921	7080
3181	7050
9421	7035
6940	7034
3220	7031
9001	7025
9520	7020
9530	7009
2750	7004
9046	7006
1280	7002
9320	7000
9571	6150
2900	5950
9181	5535
9500	5532
2053	5395
2141	7500
2205	7100
2800	7560
2730	7550
3230	7224
3300	7217
3301	7491
3350	7950

9300	7595
9252	7235
9410	7490
3053	5920
9561	7205
3062	5500
3080	7096
3150	7150
2276	7496
9008	7170
3030	2575
6838	7211
9430	5530
9220	7180
9570	1313
9400	2383
9560	
9040	

[a] Two images (7031 and 7004) were presented twice in the sequence
